# A redetermination of (2-methoxy­phen­yl)diphenyl­phosphine

**DOI:** 10.1107/S1600536809038835

**Published:** 2009-10-07

**Authors:** Omar bin Shawkataly, Mohd. Aslam A. Pankhi, Imthyaz Ahmed Khan, Chin Sing Yeap, Hoong-Kun Fun

**Affiliations:** a Chemical Sciences Programme, Centre for Distance Education, Universiti Sains Malaysia, 11800 USM, Penang, Malaysia; b X-ray Crystallography Unit, School of Physics, Universiti Sains Malaysia, 11800 USM, Penang, Malaysia

## Abstract

The asymmetric unit of the title triphenyl­phosphine compound, C_19_H_17_OP, consists of two crystallographically independent mol­ecules with similar conformations. One of these two mol­ecules has a whole-mol­ecule disorder over two positions with refined occupancies of 0.753 (3) and 0.247 (3). The dihedral angles between the three benzene rings are 89.69 (7), 76.54 (7) and 86.02 (7)° in the non-disordered mol­ecule and the corresponding angles are 88.3 (4), 83.2 (4) and 84.2 (3)° for the major component and 80.2 (11), 89.5 (11) and 74.4 (9)° for the minor component of the disordered mol­ecule. This structure has been reported previously [Suomalainen *et al.* (2000 ▶). *Eur. J. Inorg. Chem.* pp. 2607–2613]; however, the disorder detailed here was not mentioned in that determination. In the crystal structure, the mol­ecules are stacked down the *b* axis and stabilized by C—H⋯π inter­actions.

## Related literature

For a previous report of this mol­ecule, see: Suomalainen *et al.* (2000 ▶). For P–C bond lengths and C–P–C angles in related structures, see: Dunne & Orpen (1991 ▶); Shawkataly *et al.* (2009 ▶). For the stereochemistry of 2-methoxy­phenyl diphenyl­phosphine complexes, see: Dahlenburg *et al.* (1997 ▶); Moreno *et al.* (2005 ▶). For the stability of the temperature controller used for the data collection, see: Cosier & Glazer (1986 ▶).
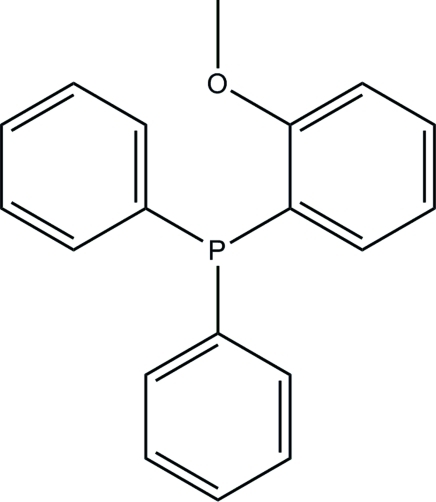


## Experimental

### 

#### Crystal data

C_19_H_17_OP
*M* *_r_* = 292.30
Monoclinic, 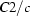

*a* = 31.1813 (8) Å
*b* = 7.1474 (2) Å
*c* = 28.3025 (8) Å
β = 90.3795 (12)°
*V* = 6307.5 (3) Å^3^
*Z* = 16
Mo *K*α radiation
μ = 0.17 mm^−1^
*T* = 100 K
0.78 × 0.24 × 0.13 mm

#### Data collection

Bruker SMART APEXII CCD area-detector diffractometer
Absorption correction: multi-scan (**SADABS**; Bruker, 2005 ▶) *T*_min_ = 0.879, *T*_max_ = 0.978
49460 measured reflections
11740 independent reflections
8873 reflections with *I* > 2σ(*I*)
*R*_int_ = 0.035

#### Refinement

*R*[*F* ^2^ > 2σ(*F* ^2^)] = 0.054
*wR*(*F* ^2^) = 0.123
*S* = 1.10
11740 reflections
566 parameters
157 restraints
H-atom parameters constrained
Δρ_max_ = 0.33 e Å^−3^
Δρ_min_ = −0.25 e Å^−3^

### 

Data collection: *APEX2* (Bruker, 2005 ▶); cell refinement: *SAINT* (Bruker, 2005 ▶); data reduction: *SAINT* (Bruker, 2005 ▶); program(s) used to solve structure: *SHELXTL* (Sheldrick, 2008 ▶); program(s) used to refine structure: *SHELXTL*; molecular graphics: *SHELXTL*; software used to prepare material for publication: *SHELXTL* and *PLATON* (Spek, 2009 ▶).

## Supplementary Material

Crystal structure: contains datablocks global, I. DOI: 10.1107/S1600536809038835/sj2649sup1.cif

Structure factors: contains datablocks I. DOI: 10.1107/S1600536809038835/sj2649Isup2.hkl

Additional supplementary materials:  crystallographic information; 3D view; checkCIF report

## Figures and Tables

**Table 1:** Hydrogen-bond geometry (Å, °)

*D*—H⋯*A*	*D*—H	H⋯*A*	*D*⋯*A*	*D*—H⋯*A*
C4*A*—H4*AA*⋯*Cg*1^i^	0.93	2.65	3.520 (9)	156
C4*A*—H4*AA*⋯*Cg*2^i^	0.93	2.58	3.471 (3)	159
C17*A*—H17*A*⋯*Cg*3^ii^	0.93	2.91	3.645 (8)	137
C10*C*—H10*C*⋯*Cg*4^ii^	0.93	2.78	3.561 (15)	142

Symmetry codes: (i) 
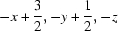
; (ii) 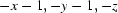. *Cg*1, *Cg*2 *Cg*3 and *Cg*4 are the centroids of the C13*C*–C18*C*, C7*B*–C12*B*, C7*C*–C12*C* and C13*A*–C18*A *benzene rings, respectively.

**Fig. 1.**
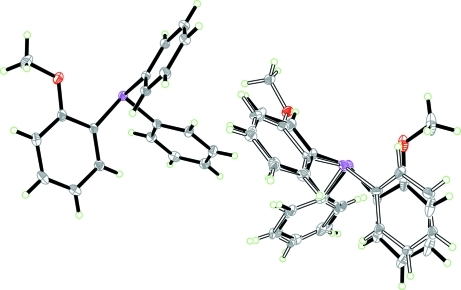
The molecular structure of the title compound with 30% probability ellipsoids for non-H atoms. Both disorder components are shown, with open bonds for the minor disorder component.

**Fig. 2.**
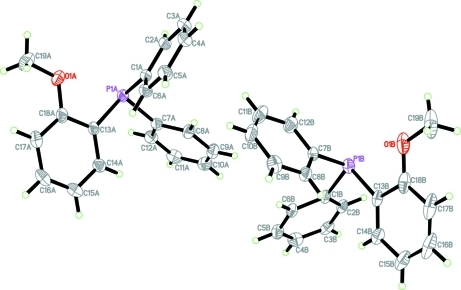
The molecular structure of the title compound with atoms label and 50% probability ellipsoids for non-H atoms. Only major disorder component is shown.

**Fig. 3.**
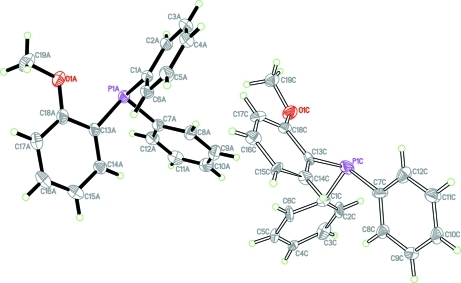
The molecular structure of the title compound with atoms label and 50% probability ellipsoids for non-H atoms. Only minor disorder component is shown.

**Fig. 4.**
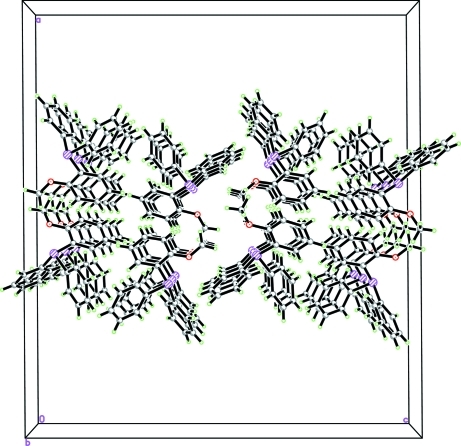
The crystal packing of the title compound, viewed down the b axis, showing the molecules stacked down the b axis. Only the major disorder component is shown.

## supplementary crystallographic information

### Comment

Substituted triphenylphosphines as well as the parent compound have found widespread use as ligands in transition-metal chemistry, notably in homogeneous catalysis. The structure of the title compound was reported by Suomalainen *et al.*, (2000) for the first time. However, the whole-molecule disorder in one of the the two molecules in the asymmetric unit present in 2-methoxyphenyl diphenylphosphine (I) has not been reported. This resulted in large residual peaks in the difference map in his solution. This prompts us to redetermine the structure. Our redetermination has taken care of these large residual peaks which are the consequence of the whole-molecule disorder as shown in molecules *B* and *C* (Fig. 1, 2 & 3).

Interestingly, the evidence of the disorder in this ligand has however been reported by Moreno *et al.*, (2005) in a ruthenium complex.

This study was taken up as part of a project to study the stereochemistry of substituted triphenylphosphine ligands.

The asymmetric unit of (I), consists of two crystallographically independent molecules, *A* and *B*/*C*, with similar conformation (Fig. 1, 2 & 3). Molecule *B*/*C* are the major/minor components of the whole-molecule disorder over two positions with refined occupancies of 0.753 (3) and 0.247 (3) respectively. The P–C bond lengths and C–P–C angles for the asymmetric unit are comparable to the related structures (Dunne & Orpen, 1991; Suomalainen *et al.*, 2000; Shawkataly *et al.*, 2009). The dihedral angles between the three benzene rings [C1A–C6A/C7A–C12A, C1A–C6A/C13A–C18A and C7A–C13A/C13A–C18A] are 89.69 (7), 76.54 (7) and 86.02 (7)° in molecule *A* and the corresponding angles for major *B* and minor *C* components of the whole-molecule are 88.3 (4), 83.2 (4), 84.2 (3)° and 80.2 (11), 89.5 (11), 74.4 (9)°, respectively.

In the crystal structure, the molecules are stacked down the *b* axis (Fig. 4) and stabilized by the C—H···π interactions (Table 1).

### Experimental

The title compound was supplied by Strem Chemicals. Single crystals of (I) were obtained by slow evaporation of ethanol solution.

### Refinement

All hydrogen atoms were positioned geometrically and refined using a riding model with C—H = 0.93–0.96 Å and *U*_iso_(H) = 1.2 or 1.5 *U*_eq_(C). A rotating-group model was applied for methyl groups. The same U^ij^ parameters were used for atom pair C14B/C14C and all disordered atoms were subjected to rigid bond restraints (SAME and DELU). The *C* molecule is statistically disordered about the inversion center (3/4, 3/4, 0) with no actual close contacts (*i.e.* given a *C* molecule, the opposite molecule is a *B* molecule).

### Crystal data

**Table:**

C_19_H_17_OP	*F*(000) = 2464
*M**_r_* = 292.30	*D*_x_ = 1.231 Mg m^−^^3^
Monoclinic, *C*2/*c*	Mo *K*α radiation, λ = 0.71073 Å
Hall symbol: -C 2yc	Cell parameters from 9973 reflections
*a* = 31.1813 (8) Å	θ = 2.9–31.4°
*b* = 7.1474 (2) Å	µ = 0.17 mm^−^^1^
*c* = 28.3025 (8) Å	*T* = 100 K
β = 90.3795 (12)°	Needle, colourless
*V* = 6307.5 (3) Å^3^	0.78 × 0.24 × 0.13 mm
*Z* = 16	

### Data collection

**Table:**

Bruker SMART APEXII CCD area-detector diffractometer	11740 independent reflections
Radiation source: fine-focus sealed tube	8873 reflections with *I* > 2σ(*I*)
graphite	*R*_int_ = 0.035
φ and ω scans	θ_max_ = 33.0°, θ_min_ = 1.4°
Absorption correction: multi-scan (*SADABS*; Bruker, 2005)	*h* = −47→47
*T*_min_ = 0.879, *T*_max_ = 0.978	*k* = −10→10
49460 measured reflections	*l* = −42→43

### Refinement

**Table:**

Refinement on *F*^2^	Primary atom site location: structure-invariant direct methods
Least-squares matrix: full	Secondary atom site location: difference Fourier map
*R*[*F*^2^ > 2σ(*F*^2^)] = 0.054	Hydrogen site location: inferred from neighbouring sites
*wR*(*F*^2^) = 0.123	H-atom parameters constrained
*S* = 1.10	*w* = 1/[σ^2^(*F*_o_^2^) + (0.0294*P*)^2^ + 8.0384*P*] where *P* = (*F*_o_^2^ + 2*F*_c_^2^)/3
11740 reflections	(Δ/σ)_max_ = 0.001
566 parameters	Δρ_max_ = 0.33 e Å^−^^3^
157 restraints	Δρ_min_ = −0.25 e Å^−^^3^

### Special details

**Table:**

Experimental. The crystal was placed in the cold stream of an Oxford Cyrosystems Cobra open-flow nitrogen cryostat (Cosier & Glazer, 1986) operating at 100.0 (1) K.
Geometry. All e.s.d.'s (except the e.s.d. in the dihedral angle between two l.s. planes) are estimated using the full covariance matrix. The cell e.s.d.'s are taken into account individually in the estimation of e.s.d.'s in distances, angles and torsion angles; correlations between e.s.d.'s in cell parameters are only used when they are defined by crystal symmetry. An approximate (isotropic) treatment of cell e.s.d.'s is used for estimating e.s.d.'s involving l.s. planes.
Refinement. Refinement of *F*^2^ against ALL reflections. The weighted *R*-factor *wR* and goodness of fit *S* are based on *F*^2^, conventional *R*-factors *R* are based on *F*, with *F* set to zero for negative *F*^2^. The threshold expression of *F*^2^ > σ(*F*^2^) is used only for calculating *R*-factors(gt) *etc*. and is not relevant to the choice of reflections for refinement. *R*-factors based on *F*^2^ are statistically about twice as large as those based on *F*, and *R*- factors based on ALL data will be even larger.

### Fractional atomic coordinates and isotropic or equivalent isotropic displacement parameters (Å^2^)

**Table:**

	*x*	*y*	*z*	*U*_iso_*/*U*_eq_	Occ. (<1)
P1A	0.922433 (11)	0.68949 (6)	0.081792 (13)	0.02379 (8)	
O1A	0.98898 (3)	0.42835 (19)	0.06278 (4)	0.0356 (3)	
C1A	0.89016 (4)	0.5270 (2)	0.04611 (5)	0.0227 (3)	
C2A	0.87439 (4)	0.5889 (2)	0.00231 (5)	0.0268 (3)	
H2AA	0.8805	0.7097	−0.0078	0.032*	
C3A	0.84983 (5)	0.4716 (3)	−0.02601 (5)	0.0311 (3)	
H3AA	0.8393	0.5148	−0.0548	0.037*	
C4A	0.84080 (5)	0.2902 (3)	−0.01165 (5)	0.0309 (3)	
H4AA	0.8245	0.2116	−0.0309	0.037*	
C5A	0.85617 (5)	0.2268 (2)	0.03158 (5)	0.0293 (3)	
H5AA	0.8501	0.1057	0.0415	0.035*	
C6A	0.88069 (4)	0.3442 (2)	0.06009 (5)	0.0256 (3)	
H6AA	0.8910	0.3004	0.0890	0.031*	
C7A	0.88118 (4)	0.8042 (2)	0.11748 (5)	0.0236 (3)	
C8A	0.83846 (5)	0.7462 (2)	0.11895 (5)	0.0274 (3)	
H8AA	0.8300	0.6403	0.1023	0.033*	
C9A	0.80846 (5)	0.8451 (2)	0.14506 (6)	0.0312 (3)	
H9AA	0.7800	0.8065	0.1452	0.037*	
C10A	0.82081 (5)	1.0009 (2)	0.17091 (6)	0.0332 (3)	
H10A	0.8008	1.0657	0.1888	0.040*	
C11A	0.86316 (6)	1.0596 (2)	0.17001 (6)	0.0339 (3)	
H11A	0.8716	1.1638	0.1874	0.041*	
C12A	0.89293 (5)	0.9637 (2)	0.14327 (5)	0.0291 (3)	
H12A	0.9211	1.0057	0.1424	0.035*	
C13A	0.94579 (4)	0.5263 (2)	0.12501 (5)	0.0235 (3)	
C14A	0.93348 (5)	0.5143 (2)	0.17235 (5)	0.0264 (3)	
H14A	0.9118	0.5918	0.1835	0.032*	
C15A	0.95316 (5)	0.3878 (2)	0.20307 (5)	0.0317 (3)	
H15A	0.9447	0.3811	0.2345	0.038*	
C16A	0.98528 (5)	0.2724 (2)	0.18670 (6)	0.0334 (3)	
H16A	0.9984	0.1881	0.2072	0.040*	
C17A	0.99830 (5)	0.2808 (2)	0.13978 (6)	0.0311 (3)	
H17A	1.0199	0.2024	0.1289	0.037*	
C18A	0.97864 (4)	0.4080 (2)	0.10933 (5)	0.0277 (3)	
C19A	1.01988 (7)	0.3008 (4)	0.04412 (7)	0.0544 (6)	
H19A	1.0236	0.3246	0.0110	0.082*	
H19B	1.0100	0.1748	0.0485	0.082*	
H19C	1.0468	0.3175	0.0603	0.082*	
P1B	0.63959 (15)	0.6772 (5)	0.13064 (12)	0.0268 (4)	0.753 (3)
O1B	0.58298 (5)	0.4273 (3)	0.08223 (7)	0.0445 (5)	0.753 (3)
C1B	0.6620 (3)	0.7991 (12)	0.1822 (3)	0.0232 (12)	0.753 (3)
C2B	0.6393 (3)	0.9541 (11)	0.1994 (3)	0.0254 (9)	0.753 (3)
H2BA	0.6142	0.9922	0.1844	0.031*	0.753 (3)
C3B	0.6543 (3)	1.0510 (12)	0.2388 (3)	0.0308 (11)	0.753 (3)
H3BA	0.6386	1.1506	0.2508	0.037*	0.753 (3)
C4B	0.6923 (2)	0.9991 (11)	0.2598 (3)	0.0334 (15)	0.753 (3)
H4BA	0.7029	1.0674	0.2853	0.040*	0.753 (3)
C5B	0.7149 (3)	0.8462 (12)	0.2434 (3)	0.0325 (15)	0.753 (3)
H5BA	0.7404	0.8115	0.2583	0.039*	0.753 (3)
C6B	0.7000 (3)	0.7437 (12)	0.2049 (3)	0.0233 (9)	0.753 (3)
H6BA	0.7151	0.6396	0.1943	0.028*	0.753 (3)
C7B	0.68283 (18)	0.5167 (9)	0.1152 (3)	0.0244 (11)	0.753 (3)
C8B	0.68720 (17)	0.3390 (7)	0.1351 (2)	0.0257 (7)	0.753 (3)
H8BA	0.6667	0.2953	0.1562	0.031*	0.753 (3)
C9B	0.7221 (2)	0.2265 (10)	0.1235 (3)	0.0352 (14)	0.753 (3)
H9BA	0.7247	0.1077	0.1366	0.042*	0.753 (3)
C10B	0.7534 (2)	0.2906 (9)	0.0922 (3)	0.0375 (14)	0.753 (3)
H10B	0.7765	0.2152	0.0844	0.045*	0.753 (3)
C11B	0.7493 (2)	0.4669 (8)	0.0733 (3)	0.0383 (12)	0.753 (3)
H11B	0.7703	0.5117	0.0530	0.046*	0.753 (3)
C12B	0.71416 (17)	0.5798 (9)	0.0841 (3)	0.0337 (13)	0.753 (3)
H12B	0.7116	0.6977	0.0705	0.040*	0.753 (3)
C13B	0.60176 (18)	0.5166 (9)	0.16012 (18)	0.0257 (9)	0.753 (3)
C14B	0.5956 (2)	0.4999 (9)	0.20859 (17)	0.0304 (10)	0.753 (3)
H14B	0.6130	0.5685	0.2291	0.037*	0.753 (3)
C15B	0.56431 (18)	0.3837 (8)	0.22724 (15)	0.0410 (10)	0.753 (3)
H15B	0.5598	0.3795	0.2597	0.049*	0.753 (3)
C16B	0.54009 (16)	0.2750 (8)	0.19734 (18)	0.0511 (12)	0.753 (3)
H16B	0.5197	0.1945	0.2099	0.061*	0.753 (3)
C17B	0.54545 (16)	0.2829 (8)	0.14911 (18)	0.0481 (12)	0.753 (3)
H17B	0.5290	0.2073	0.1293	0.058*	0.753 (3)
C18B	0.5757 (2)	0.4047 (11)	0.13005 (18)	0.0381 (11)	0.753 (3)
C19B	0.55769 (8)	0.3189 (5)	0.04961 (13)	0.0667 (11)	0.753 (3)
H19D	0.5654	0.3508	0.0178	0.100*	0.753 (3)
H19E	0.5278	0.3453	0.0543	0.100*	0.753 (3)
H19F	0.5629	0.1882	0.0549	0.100*	0.753 (3)
P1C	0.6453 (5)	0.6726 (14)	0.1308 (4)	0.0283 (15)	0.247 (3)
O1C	0.72148 (13)	0.7423 (6)	0.07269 (15)	0.0252 (10)	0.247 (3)
C1C	0.6668 (8)	0.781 (3)	0.1856 (8)	0.018 (2)	0.247 (3)
C2C	0.6454 (9)	0.937 (4)	0.2032 (10)	0.030 (3)	0.247 (3)
H2CA	0.6199	0.9740	0.1888	0.037*	0.247 (3)
C3C	0.6608 (8)	1.040 (4)	0.2415 (10)	0.031 (4)	0.247 (3)
H3CA	0.6463	1.1472	0.2509	0.037*	0.247 (3)
C4C	0.6969 (6)	0.985 (3)	0.2652 (7)	0.022 (2)	0.247 (3)
H4CA	0.7053	1.0434	0.2931	0.027*	0.247 (3)
C5C	0.7207 (7)	0.839 (3)	0.2466 (8)	0.017 (2)	0.247 (3)
H5CA	0.7469	0.8069	0.2602	0.021*	0.247 (3)
C6C	0.7054 (9)	0.741 (4)	0.2080 (11)	0.029 (3)	0.247 (3)
H6CA	0.7220	0.6432	0.1963	0.035*	0.247 (3)
C7C	0.6038 (7)	0.515 (3)	0.1504 (5)	0.027 (3)	0.247 (3)
C8C	0.5923 (5)	0.512 (2)	0.1981 (4)	0.0179 (18)	0.247 (3)
H8CA	0.6050	0.5937	0.2195	0.022*	0.247 (3)
C9C	0.5611 (6)	0.381 (3)	0.2131 (3)	0.027 (2)	0.247 (3)
H9CA	0.5552	0.3687	0.2451	0.032*	0.247 (3)
C10C	0.5388 (5)	0.270 (2)	0.1800 (3)	0.027 (2)	0.247 (3)
H10C	0.5174	0.1875	0.1895	0.033*	0.247 (3)
C11C	0.5496 (5)	0.286 (3)	0.1329 (3)	0.029 (2)	0.247 (3)
H11C	0.5348	0.2158	0.1105	0.035*	0.247 (3)
C12C	0.5818 (6)	0.404 (3)	0.1183 (4)	0.026 (3)	0.247 (3)
H12C	0.5888	0.4101	0.0864	0.031*	0.247 (3)
C13C	0.6904 (5)	0.507 (3)	0.1199 (8)	0.020 (2)	0.247 (3)
C14C	0.6915 (6)	0.321 (3)	0.1361 (9)	0.0304 (10)	0.247 (3)
H14C	0.6696	0.2816	0.1560	0.037*	0.247 (3)
C15C	0.7225 (6)	0.196 (3)	0.1247 (8)	0.019 (2)	0.247 (3)
H15C	0.7226	0.0753	0.1372	0.023*	0.247 (3)
C16C	0.7535 (7)	0.254 (3)	0.0939 (9)	0.024 (2)	0.247 (3)
H16C	0.7755	0.1715	0.0863	0.029*	0.247 (3)
C17C	0.7538 (6)	0.428 (2)	0.0736 (6)	0.024 (2)	0.247 (3)
H17C	0.7740	0.4585	0.0509	0.028*	0.247 (3)
C18C	0.7232 (5)	0.559 (2)	0.0877 (6)	0.020 (2)	0.247 (3)
C19C	0.74898 (19)	0.7931 (10)	0.0343 (2)	0.0293 (14)	0.247 (3)
H19I	0.7424	0.9182	0.0243	0.044*	0.247 (3)
H19G	0.7445	0.7082	0.0085	0.044*	0.247 (3)
H19H	0.7784	0.7868	0.0446	0.044*	0.247 (3)

### Atomic displacement parameters (Å^2^)

**Table:**

	*U*^11^	*U*^22^	*U*^33^	*U*^12^	*U*^13^	*U*^23^
P1A	0.01805 (15)	0.02824 (19)	0.02511 (16)	−0.00272 (14)	0.00114 (12)	0.00586 (15)
O1A	0.0244 (5)	0.0477 (7)	0.0347 (6)	0.0082 (5)	0.0081 (4)	0.0068 (5)
C1A	0.0180 (5)	0.0287 (7)	0.0215 (6)	0.0011 (5)	0.0010 (4)	0.0040 (5)
C2A	0.0230 (6)	0.0322 (8)	0.0252 (6)	0.0035 (6)	0.0004 (5)	0.0077 (6)
C3A	0.0278 (7)	0.0425 (9)	0.0229 (6)	0.0034 (7)	−0.0034 (5)	0.0055 (6)
C4A	0.0262 (7)	0.0401 (9)	0.0262 (7)	−0.0003 (6)	−0.0044 (5)	−0.0028 (6)
C5A	0.0270 (7)	0.0302 (8)	0.0306 (7)	−0.0014 (6)	−0.0019 (6)	0.0034 (6)
C6A	0.0235 (6)	0.0307 (8)	0.0226 (6)	−0.0021 (6)	−0.0015 (5)	0.0066 (6)
C7A	0.0225 (6)	0.0234 (7)	0.0249 (6)	−0.0013 (5)	−0.0005 (5)	0.0064 (5)
C8A	0.0232 (6)	0.0263 (7)	0.0328 (7)	−0.0021 (6)	0.0020 (5)	0.0013 (6)
C9A	0.0234 (6)	0.0319 (8)	0.0383 (8)	0.0021 (6)	0.0034 (6)	0.0018 (7)
C10A	0.0380 (8)	0.0301 (8)	0.0316 (7)	0.0074 (7)	0.0036 (6)	0.0020 (7)
C11A	0.0442 (9)	0.0266 (8)	0.0309 (7)	−0.0016 (7)	−0.0037 (6)	−0.0006 (6)
C12A	0.0295 (7)	0.0279 (8)	0.0298 (7)	−0.0060 (6)	−0.0024 (6)	0.0047 (6)
C13A	0.0164 (5)	0.0286 (7)	0.0255 (6)	−0.0030 (5)	−0.0027 (5)	0.0019 (6)
C14A	0.0254 (6)	0.0279 (7)	0.0258 (6)	−0.0020 (6)	−0.0027 (5)	0.0003 (6)
C15A	0.0383 (8)	0.0308 (8)	0.0260 (7)	−0.0020 (7)	−0.0085 (6)	0.0020 (6)
C16A	0.0359 (8)	0.0296 (8)	0.0345 (8)	−0.0017 (7)	−0.0158 (6)	0.0035 (7)
C17A	0.0220 (6)	0.0319 (8)	0.0395 (8)	0.0017 (6)	−0.0063 (6)	0.0006 (7)
C18A	0.0172 (6)	0.0338 (8)	0.0320 (7)	−0.0028 (6)	−0.0019 (5)	0.0024 (6)
C19A	0.0431 (10)	0.0717 (16)	0.0486 (11)	0.0230 (11)	0.0186 (8)	0.0086 (11)
P1B	0.0223 (7)	0.0251 (6)	0.0331 (7)	−0.0009 (4)	−0.0026 (4)	0.0016 (5)
O1B	0.0230 (7)	0.0508 (11)	0.0596 (12)	−0.0003 (7)	−0.0055 (7)	−0.0228 (9)
C1B	0.0171 (14)	0.019 (2)	0.033 (2)	0.0003 (12)	0.0018 (11)	0.0087 (14)
C2B	0.022 (3)	0.0197 (15)	0.0346 (18)	0.0056 (14)	0.0058 (14)	0.0072 (15)
C3B	0.032 (3)	0.0228 (18)	0.0381 (19)	0.0035 (15)	0.0141 (16)	0.0051 (14)
C4B	0.042 (3)	0.0262 (19)	0.032 (2)	−0.0050 (17)	0.0038 (16)	−0.0053 (18)
C5B	0.024 (3)	0.037 (2)	0.036 (2)	−0.0034 (16)	−0.0011 (16)	0.0020 (16)
C6B	0.020 (2)	0.0192 (17)	0.0303 (19)	−0.0017 (14)	0.0025 (14)	−0.0032 (15)
C7B	0.0172 (18)	0.0297 (15)	0.026 (2)	−0.0052 (12)	−0.0023 (17)	−0.0002 (12)
C8B	0.0198 (15)	0.0315 (17)	0.0257 (11)	0.0019 (10)	0.0014 (11)	−0.0023 (12)
C9B	0.033 (2)	0.032 (3)	0.040 (3)	0.0024 (16)	−0.0053 (16)	−0.0005 (18)
C10B	0.0193 (15)	0.048 (4)	0.045 (3)	0.000 (2)	0.0028 (14)	−0.012 (2)
C11B	0.0277 (19)	0.040 (3)	0.047 (2)	−0.0135 (18)	0.0116 (15)	−0.0087 (19)
C12B	0.029 (2)	0.0335 (19)	0.038 (2)	−0.0073 (14)	0.0024 (18)	−0.0023 (15)
C13B	0.0163 (11)	0.0194 (13)	0.042 (3)	−0.0016 (9)	0.0003 (16)	0.0011 (18)
C14B	0.0233 (15)	0.0258 (14)	0.042 (2)	0.0020 (11)	−0.0002 (15)	0.0160 (17)
C15B	0.0272 (16)	0.0299 (16)	0.066 (3)	0.0030 (12)	0.017 (2)	0.016 (2)
C16B	0.0222 (15)	0.0285 (16)	0.103 (4)	−0.0010 (12)	0.017 (2)	0.009 (3)
C17B	0.0208 (12)	0.0288 (17)	0.095 (4)	−0.0038 (10)	0.003 (3)	−0.009 (4)
C18B	0.0203 (16)	0.0334 (18)	0.061 (3)	0.0038 (12)	0.000 (2)	−0.010 (2)
C19B	0.0240 (11)	0.079 (2)	0.097 (2)	0.0046 (13)	−0.0092 (13)	−0.057 (2)
P1C	0.031 (4)	0.0269 (19)	0.0274 (18)	−0.0050 (17)	−0.0063 (16)	0.0141 (16)
O1C	0.025 (2)	0.024 (2)	0.027 (2)	−0.0006 (16)	0.0063 (15)	0.0068 (17)
C1C	0.026 (7)	0.008 (4)	0.019 (4)	0.000 (4)	0.005 (4)	0.001 (4)
C2C	0.014 (5)	0.033 (7)	0.043 (5)	0.011 (3)	−0.001 (3)	0.017 (4)
C3C	0.027 (6)	0.021 (6)	0.044 (7)	0.004 (4)	0.009 (4)	−0.012 (5)
C4C	0.013 (3)	0.030 (5)	0.024 (4)	−0.003 (3)	0.005 (3)	0.008 (3)
C5C	0.012 (4)	0.012 (4)	0.028 (5)	0.000 (3)	−0.001 (3)	−0.009 (3)
C6C	0.012 (4)	0.037 (7)	0.039 (6)	0.004 (4)	−0.007 (3)	0.006 (4)
C7C	0.032 (5)	0.038 (5)	0.013 (4)	0.004 (3)	0.004 (3)	0.004 (3)
C8C	0.012 (3)	0.019 (4)	0.023 (4)	0.001 (2)	−0.004 (3)	0.013 (3)
C9C	0.028 (4)	0.033 (4)	0.019 (3)	0.002 (3)	0.001 (3)	0.007 (4)
C10C	0.022 (4)	0.027 (4)	0.034 (4)	−0.009 (3)	−0.002 (4)	0.003 (5)
C11C	0.034 (6)	0.031 (4)	0.022 (3)	−0.004 (4)	−0.002 (3)	0.000 (4)
C12C	0.026 (6)	0.023 (4)	0.029 (5)	−0.009 (4)	0.005 (3)	−0.003 (4)
C13C	0.017 (5)	0.023 (4)	0.019 (4)	−0.001 (3)	−0.009 (4)	−0.004 (3)
C14C	0.0233 (15)	0.0258 (14)	0.042 (2)	0.0020 (11)	−0.0002 (15)	0.0160 (17)
C15C	0.012 (4)	0.023 (5)	0.023 (5)	0.001 (3)	0.009 (3)	0.008 (4)
C16C	0.030 (5)	0.017 (4)	0.025 (5)	−0.006 (3)	−0.001 (3)	0.005 (4)
C17C	0.030 (5)	0.025 (5)	0.016 (3)	−0.001 (3)	0.006 (3)	0.000 (3)
C18C	0.024 (5)	0.020 (4)	0.017 (3)	−0.011 (3)	−0.002 (4)	0.006 (3)
C19C	0.026 (3)	0.035 (3)	0.026 (3)	0.001 (2)	0.005 (2)	0.010 (2)

### Geometric parameters (Å, °)

**Table:**

P1A—C7A	1.8343 (15)	C9B—C10B	1.396 (7)
P1A—C1A	1.8351 (15)	C9B—H9BA	0.9300
P1A—C13A	1.8370 (15)	C10B—C11B	1.375 (6)
O1A—C18A	1.3661 (18)	C10B—H10B	0.9300
O1A—C19A	1.430 (2)	C11B—C12B	1.397 (6)
C1A—C6A	1.397 (2)	C11B—H11B	0.9300
C1A—C2A	1.4021 (19)	C12B—H12B	0.9300
C2A—C3A	1.387 (2)	C13B—C14B	1.392 (6)
C2A—H2AA	0.9300	C13B—C18B	1.418 (6)
C3A—C4A	1.389 (2)	C14B—C15B	1.389 (6)
C3A—H3AA	0.9300	C14B—H14B	0.9300
C4A—C5A	1.387 (2)	C15B—C16B	1.372 (6)
C4A—H4AA	0.9300	C15B—H15B	0.9300
C5A—C6A	1.390 (2)	C16B—C17B	1.378 (6)
C5A—H5AA	0.9300	C16B—H16B	0.9300
C6A—H6AA	0.9300	C17B—C18B	1.396 (6)
C7A—C8A	1.3957 (19)	C17B—H17B	0.9300
C7A—C12A	1.401 (2)	C19B—H19D	0.9600
C8A—C9A	1.389 (2)	C19B—H19E	0.9600
C8A—H8AA	0.9300	C19B—H19F	0.9600
C9A—C10A	1.386 (2)	P1C—C7C	1.807 (15)
C9A—H9AA	0.9300	P1C—C1C	1.855 (12)
C10A—C11A	1.386 (2)	P1C—C13C	1.864 (15)
C10A—H10A	0.9300	O1C—C18C	1.381 (13)
C11A—C12A	1.383 (2)	O1C—C19C	1.434 (7)
C11A—H11A	0.9300	C1C—C6C	1.386 (14)
C12A—H12A	0.9300	C1C—C2C	1.394 (13)
C13A—C14A	1.399 (2)	C2C—C3C	1.390 (14)
C13A—C18A	1.403 (2)	C2C—H2CA	0.9300
C14A—C15A	1.394 (2)	C3C—C4C	1.367 (14)
C14A—H14A	0.9300	C3C—H3CA	0.9300
C15A—C16A	1.380 (2)	C4C—C5C	1.385 (13)
C15A—H15A	0.9300	C4C—H4CA	0.9300
C16A—C17A	1.392 (2)	C5C—C6C	1.381 (14)
C16A—H16A	0.9300	C5C—H5CA	0.9300
C17A—C18A	1.392 (2)	C6C—H6CA	0.9300
C17A—H17A	0.9300	C7C—C12C	1.382 (16)
C19A—H19A	0.9600	C7C—C8C	1.399 (14)
C19A—H19B	0.9600	C8C—C9C	1.416 (16)
C19A—H19C	0.9600	C8C—H8CA	0.9300
P1B—C7B	1.825 (6)	C9C—C10C	1.410 (13)
P1B—C1B	1.834 (5)	C9C—H9CA	0.9300
P1B—C13B	1.849 (5)	C10C—C11C	1.382 (10)
O1B—C18B	1.383 (5)	C10C—H10C	0.9300
O1B—C19B	1.437 (3)	C11C—C12C	1.376 (14)
C1B—C6B	1.402 (5)	C11C—H11C	0.9300
C1B—C2B	1.403 (5)	C12C—H12C	0.9300
C2B—C3B	1.389 (5)	C13C—C14C	1.407 (17)
C2B—H2BA	0.9300	C13C—C18C	1.424 (16)
C3B—C4B	1.375 (6)	C14C—C15C	1.359 (17)
C3B—H3BA	0.9300	C14C—H14C	0.9300
C4B—C5B	1.383 (6)	C15C—C16C	1.373 (16)
C4B—H4BA	0.9300	C15C—H15C	0.9300
C5B—C6B	1.392 (5)	C16C—C17C	1.368 (15)
C5B—H5BA	0.9300	C16C—H16C	0.9300
C6B—H6BA	0.9300	C17C—C18C	1.393 (14)
C7B—C12B	1.395 (7)	C17C—H17C	0.9300
C7B—C8B	1.396 (6)	C19C—H19I	0.9600
C8B—C9B	1.395 (6)	C19C—H19G	0.9600
C8B—H8BA	0.9300	C19C—H19H	0.9600
			
C7A—P1A—C1A	101.65 (6)	C8B—C9B—H9BA	119.7
C7A—P1A—C13A	101.15 (6)	C10B—C9B—H9BA	119.7
C1A—P1A—C13A	100.27 (7)	C11B—C10B—C9B	119.0 (6)
C18A—O1A—C19A	116.89 (14)	C11B—C10B—H10B	120.5
C6A—C1A—C2A	118.15 (13)	C9B—C10B—H10B	120.5
C6A—C1A—P1A	123.50 (10)	C10B—C11B—C12B	121.0 (5)
C2A—C1A—P1A	118.35 (12)	C10B—C11B—H11B	119.5
C3A—C2A—C1A	120.62 (15)	C12B—C11B—H11B	119.5
C3A—C2A—H2AA	119.7	C7B—C12B—C11B	120.3 (5)
C1A—C2A—H2AA	119.7	C7B—C12B—H12B	119.8
C2A—C3A—C4A	120.51 (14)	C11B—C12B—H12B	119.8
C2A—C3A—H3AA	119.7	C14B—C13B—C18B	117.5 (4)
C4A—C3A—H3AA	119.7	C14B—C13B—P1B	126.2 (4)
C5A—C4A—C3A	119.58 (15)	C18B—C13B—P1B	116.3 (4)
C5A—C4A—H4AA	120.2	C15B—C14B—C13B	121.8 (5)
C3A—C4A—H4AA	120.2	C15B—C14B—H14B	119.1
C4A—C5A—C6A	120.03 (15)	C13B—C14B—H14B	119.1
C4A—C5A—H5AA	120.0	C16B—C15B—C14B	119.3 (4)
C6A—C5A—H5AA	120.0	C16B—C15B—H15B	120.3
C5A—C6A—C1A	121.11 (13)	C14B—C15B—H15B	120.3
C5A—C6A—H6AA	119.4	C15B—C16B—C17B	121.2 (4)
C1A—C6A—H6AA	119.4	C15B—C16B—H16B	119.4
C8A—C7A—C12A	118.18 (14)	C17B—C16B—H16B	119.4
C8A—C7A—P1A	123.82 (12)	C16B—C17B—C18B	119.7 (4)
C12A—C7A—P1A	117.94 (11)	C16B—C17B—H17B	120.2
C9A—C8A—C7A	120.74 (15)	C18B—C17B—H17B	120.2
C9A—C8A—H8AA	119.6	O1B—C18B—C17B	124.5 (4)
C7A—C8A—H8AA	119.6	O1B—C18B—C13B	115.2 (4)
C10A—C9A—C8A	120.25 (15)	C17B—C18B—C13B	120.4 (4)
C10A—C9A—H9AA	119.9	C7C—P1C—C1C	105.1 (12)
C8A—C9A—H9AA	119.9	C7C—P1C—C13C	101.4 (10)
C9A—C10A—C11A	119.68 (15)	C1C—P1C—C13C	97.7 (10)
C9A—C10A—H10A	120.2	C18C—O1C—C19C	116.7 (7)
C11A—C10A—H10A	120.2	C6C—C1C—C2C	114.7 (12)
C12A—C11A—C10A	120.18 (15)	C6C—C1C—P1C	127.2 (13)
C12A—C11A—H11A	119.9	C2C—C1C—P1C	117.5 (13)
C10A—C11A—H11A	119.9	C3C—C2C—C1C	122.6 (14)
C11A—C12A—C7A	120.94 (14)	C3C—C2C—H2CA	118.7
C11A—C12A—H12A	119.5	C1C—C2C—H2CA	118.7
C7A—C12A—H12A	119.5	C4C—C3C—C2C	120.6 (15)
C14A—C13A—C18A	118.12 (13)	C4C—C3C—H3CA	119.7
C14A—C13A—P1A	124.53 (11)	C2C—C3C—H3CA	119.7
C18A—C13A—P1A	117.34 (11)	C3C—C4C—C5C	118.1 (15)
C15A—C14A—C13A	121.01 (15)	C3C—C4C—H4CA	120.9
C15A—C14A—H14A	119.5	C5C—C4C—H4CA	120.9
C13A—C14A—H14A	119.5	C6C—C5C—C4C	120.1 (16)
C16A—C15A—C14A	119.70 (15)	C6C—C5C—H5CA	120.0
C16A—C15A—H15A	120.2	C4C—C5C—H5CA	120.0
C14A—C15A—H15A	120.2	C5C—C6C—C1C	123.4 (16)
C15A—C16A—C17A	120.77 (14)	C5C—C6C—H6CA	118.3
C15A—C16A—H16A	119.6	C1C—C6C—H6CA	118.3
C17A—C16A—H16A	119.6	C12C—C7C—C8C	119.7 (12)
C18A—C17A—C16A	119.24 (15)	C12C—C7C—P1C	120.6 (10)
C18A—C17A—H17A	120.4	C8C—C7C—P1C	119.6 (11)
C16A—C17A—H17A	120.4	C7C—C8C—C9C	118.7 (12)
O1A—C18A—C17A	124.11 (14)	C7C—C8C—H8CA	120.6
O1A—C18A—C13A	114.73 (13)	C9C—C8C—H8CA	120.6
C17A—C18A—C13A	121.16 (14)	C10C—C9C—C8C	120.7 (10)
O1A—C19A—H19A	109.5	C10C—C9C—H9CA	119.7
O1A—C19A—H19B	109.5	C8C—C9C—H9CA	119.7
H19A—C19A—H19B	109.5	C11C—C10C—C9C	118.0 (11)
O1A—C19A—H19C	109.5	C11C—C10C—H10C	121.0
H19A—C19A—H19C	109.5	C9C—C10C—H10C	121.0
H19B—C19A—H19C	109.5	C12C—C11C—C10C	121.8 (12)
C7B—P1B—C1B	102.2 (4)	C12C—C11C—H11C	119.1
C7B—P1B—C13B	101.1 (3)	C10C—C11C—H11C	119.1
C1B—P1B—C13B	100.2 (4)	C11C—C12C—C7C	120.8 (11)
C18B—O1B—C19B	118.3 (3)	C11C—C12C—H12C	119.6
C6B—C1B—C2B	119.4 (4)	C7C—C12C—H12C	119.6
C6B—C1B—P1B	123.1 (4)	C14C—C13C—C18C	115.9 (13)
C2B—C1B—P1B	117.5 (4)	C14C—C13C—P1C	124.2 (12)
C3B—C2B—C1B	120.3 (5)	C18C—C13C—P1C	119.3 (12)
C3B—C2B—H2BA	119.8	C15C—C14C—C13C	124.2 (16)
C1B—C2B—H2BA	119.8	C15C—C14C—H14C	117.9
C4B—C3B—C2B	119.8 (5)	C13C—C14C—H14C	117.9
C4B—C3B—H3BA	120.1	C14C—C15C—C16C	117.1 (16)
C2B—C3B—H3BA	120.1	C14C—C15C—H15C	121.4
C3B—C4B—C5B	120.6 (5)	C16C—C15C—H15C	121.4
C3B—C4B—H4BA	119.7	C17C—C16C—C15C	123.2 (17)
C5B—C4B—H4BA	119.7	C17C—C16C—H16C	118.4
C4B—C5B—C6B	120.7 (5)	C15C—C16C—H16C	118.4
C4B—C5B—H5BA	119.7	C16C—C17C—C18C	119.0 (14)
C6B—C5B—H5BA	119.7	C16C—C17C—H17C	120.5
C5B—C6B—C1B	119.2 (5)	C18C—C17C—H17C	120.5
C5B—C6B—H6BA	120.4	O1C—C18C—C17C	125.1 (12)
C1B—C6B—H6BA	120.4	O1C—C18C—C13C	114.5 (11)
C12B—C7B—C8B	118.8 (5)	C17C—C18C—C13C	120.4 (12)
C12B—C7B—P1B	118.0 (4)	O1C—C19C—H19I	109.5
C8B—C7B—P1B	123.1 (4)	O1C—C19C—H19G	109.5
C9B—C8B—C7B	120.2 (5)	H19I—C19C—H19G	109.5
C9B—C8B—H8BA	119.9	O1C—C19C—H19H	109.5
C7B—C8B—H8BA	119.9	H19I—C19C—H19H	109.5
C8B—C9B—C10B	120.6 (6)	H19G—C19C—H19H	109.5
			
C7A—P1A—C1A—C6A	−90.02 (13)	C8B—C7B—C12B—C11B	−0.3 (12)
C13A—P1A—C1A—C6A	13.75 (13)	P1B—C7B—C12B—C11B	176.2 (6)
C7A—P1A—C1A—C2A	90.47 (12)	C10B—C11B—C12B—C7B	1.4 (12)
C13A—P1A—C1A—C2A	−165.76 (11)	C7B—P1B—C13B—C14B	102.8 (7)
C6A—C1A—C2A—C3A	0.5 (2)	C1B—P1B—C13B—C14B	−1.9 (7)
P1A—C1A—C2A—C3A	−179.99 (11)	C7B—P1B—C13B—C18B	−79.4 (7)
C1A—C2A—C3A—C4A	−0.6 (2)	C1B—P1B—C13B—C18B	175.9 (6)
C2A—C3A—C4A—C5A	0.6 (2)	C18B—C13B—C14B—C15B	−2.2 (10)
C3A—C4A—C5A—C6A	−0.4 (2)	P1B—C13B—C14B—C15B	175.6 (6)
C4A—C5A—C6A—C1A	0.3 (2)	C13B—C14B—C15B—C16B	3.3 (10)
C2A—C1A—C6A—C5A	−0.3 (2)	C14B—C15B—C16B—C17B	−1.8 (9)
P1A—C1A—C6A—C5A	−179.81 (11)	C15B—C16B—C17B—C18B	−0.7 (9)
C1A—P1A—C7A—C8A	8.97 (14)	C19B—O1B—C18B—C17B	0.2 (9)
C13A—P1A—C7A—C8A	−94.10 (13)	C19B—O1B—C18B—C13B	−179.8 (5)
C1A—P1A—C7A—C12A	−168.38 (11)	C16B—C17B—C18B—O1B	−178.3 (6)
C13A—P1A—C7A—C12A	88.55 (12)	C16B—C17B—C18B—C13B	1.7 (11)
C12A—C7A—C8A—C9A	0.4 (2)	C14B—C13B—C18B—O1B	179.7 (6)
P1A—C7A—C8A—C9A	−176.94 (12)	P1B—C13B—C18B—O1B	1.6 (9)
C7A—C8A—C9A—C10A	−1.4 (2)	C14B—C13B—C18B—C17B	−0.3 (11)
C8A—C9A—C10A—C11A	1.0 (2)	P1B—C13B—C18B—C17B	−178.3 (6)
C9A—C10A—C11A—C12A	0.3 (2)	C7C—P1C—C1C—C6C	105 (3)
C10A—C11A—C12A—C7A	−1.3 (2)	C13C—P1C—C1C—C6C	1(4)
C8A—C7A—C12A—C11A	0.9 (2)	C7C—P1C—C1C—C2C	−85 (3)
P1A—C7A—C12A—C11A	178.43 (12)	C13C—P1C—C1C—C2C	171 (3)
C7A—P1A—C13A—C14A	−2.63 (14)	C6C—C1C—C2C—C3C	−3(6)
C1A—P1A—C13A—C14A	−106.80 (13)	P1C—C1C—C2C—C3C	−174 (3)
C7A—P1A—C13A—C18A	178.33 (11)	C1C—C2C—C3C—C4C	−4(6)
C1A—P1A—C13A—C18A	74.16 (12)	C2C—C3C—C4C—C5C	8(5)
C18A—C13A—C14A—C15A	−0.3 (2)	C3C—C4C—C5C—C6C	−7(4)
P1A—C13A—C14A—C15A	−179.29 (12)	C4C—C5C—C6C—C1C	0(6)
C13A—C14A—C15A—C16A	0.0 (2)	C2C—C1C—C6C—C5C	4(6)
C14A—C15A—C16A—C17A	0.0 (2)	P1C—C1C—C6C—C5C	175 (3)
C15A—C16A—C17A—C18A	0.3 (2)	C1C—P1C—C7C—C12C	−178 (2)
C19A—O1A—C18A—C17A	4.5 (2)	C13C—P1C—C7C—C12C	−76 (2)
C19A—O1A—C18A—C13A	−175.18 (16)	C1C—P1C—C7C—C8C	6(2)
C16A—C17A—C18A—O1A	179.78 (15)	C13C—P1C—C7C—C8C	107 (2)
C16A—C17A—C18A—C13A	−0.5 (2)	C12C—C7C—C8C—C9C	6(3)
C14A—C13A—C18A—O1A	−179.75 (13)	P1C—C7C—C8C—C9C	−177.2 (17)
P1A—C13A—C18A—O1A	−0.65 (17)	C7C—C8C—C9C—C10C	−6(3)
C14A—C13A—C18A—C17A	0.5 (2)	C8C—C9C—C10C—C11C	3(3)
P1A—C13A—C18A—C17A	179.62 (12)	C9C—C10C—C11C—C12C	1(3)
C7B—P1B—C1B—C6B	−9.5 (11)	C10C—C11C—C12C—C7C	−2(4)
C13B—P1B—C1B—C6B	94.3 (10)	C8C—C7C—C12C—C11C	−2(4)
C7B—P1B—C1B—C2B	170.9 (9)	P1C—C7C—C12C—C11C	−178.9 (19)
C13B—P1B—C1B—C2B	−85.3 (10)	C7C—P1C—C13C—C14C	−15 (2)
C6B—C1B—C2B—C3B	−0.5 (18)	C1C—P1C—C13C—C14C	93 (2)
P1B—C1B—C2B—C3B	179.1 (9)	C7C—P1C—C13C—C18C	156.0 (17)
C1B—C2B—C3B—C4B	2.5 (17)	C1C—P1C—C13C—C18C	−97 (2)
C2B—C3B—C4B—C5B	−2.8 (15)	C18C—C13C—C14C—C15C	3(4)
C3B—C4B—C5B—C6B	1.0 (16)	P1C—C13C—C14C—C15C	174 (2)
C4B—C5B—C6B—C1B	1.1 (17)	C13C—C14C—C15C—C16C	−3(4)
C2B—C1B—C6B—C5B	−1.3 (17)	C14C—C15C—C16C—C17C	−2(4)
P1B—C1B—C6B—C5B	179.1 (9)	C15C—C16C—C17C—C18C	6(4)
C1B—P1B—C7B—C12B	−89.0 (7)	C19C—O1C—C18C—C17C	10 (2)
C13B—P1B—C7B—C12B	167.9 (6)	C19C—O1C—C18C—C13C	−169.5 (14)
C1B—P1B—C7B—C8B	87.3 (8)	C16C—C17C—C18C—O1C	175 (2)
C13B—P1B—C7B—C8B	−15.8 (7)	C16C—C17C—C18C—C13C	−5(3)
C12B—C7B—C8B—C9B	−0.7 (12)	C14C—C13C—C18C—O1C	−179.5 (19)
P1B—C7B—C8B—C9B	−177.0 (7)	P1C—C13C—C18C—O1C	9(2)
C7B—C8B—C9B—C10B	0.7 (14)	C14C—C13C—C18C—C17C	1(3)
C8B—C9B—C10B—C11B	0.4 (15)	P1C—C13C—C18C—C17C	−170.6 (17)
C9B—C10B—C11B—C12B	−1.4 (14)		

### Hydrogen-bond geometry (Å, °)

**Table:**

*D*—H···*A*	*D*—H	H···*A*	*D*···*A*	*D*—H···*A*
C4A—H4AA···Cg1^i^	0.93	2.65	3.520 (9)	156
C4A—H4AA···Cg2^i^	0.93	2.58	3.471 (3)	159
C17A—H17A···Cg3^ii^	0.93	2.91	3.645 (8)	137
C10C—H10C···Cg4^ii^	0.93	2.78	3.561 (15)	142

Symmetry codes: (i) −*x*+3/2, −*y*+1/2, −*z*; (ii) −*x*−1, −*y*−1, −*z*.
